# Prediction of network pharmacology, molecular docking-based strategy, and vitro assays to determine potential pharmacological mechanism of *Dioscoreae bulbiferae* and *Bruceae fructus* against laryngocarcinoma

**DOI:** 10.1097/MD.0000000000036771

**Published:** 2023-12-22

**Authors:** Zhongbiao Wu, Zhongyan Zhu, Jian Cao, Weikun Wu, Chengcheng Deng, Qiang Xie, Shiping Hu

**Affiliations:** a Jiangxi Hospital of Integrated Traditional Chinese and Western Medicine, Nanchang, Jiangxi, China; b Affiliated Hospital of Jiangxi University of Traditional Chinese Medicine, Nanchang, Jiangxi, China; c Jiangxi University of Traditional Chinese Medicine, Nanchang, Jiangxi, China.

**Keywords:** *Bruceae fructus*, *Dioscoreae bulbiferae*, laryngocarcinoma, molecular docking, network pharmacology, vitro assays

## Abstract

**Background:**

Based on network pharmacology, molecular docking, and vitro assays, investigate the probable pharmacological mechanism of *Dioscoreae bulbiferae* and *Bruceae fructus* in the treatment of laryngocarcinoma.

**Methods:**

The active components and targets of *Dioscoreae bulbiferae* and *Bruceae fructus* were retrieved from the Traditional Chinese Medicine Systems Pharmacology Database and Analysis Platform database. Targets linked with laryngocarcinoma were gathered from the GeneCards, DisGeNET, and DrugBank databases. The String database was utilized to build a protein–protein interaction network of common medication and illness targets, after which the core targets were filtered out. The Metascape database served for gene ontology enrichment and Kyoto encyclopedia of genes and genomes pathway analysis of common targets. AutoDock then performed molecular docking between the essential component and the vital target. To investigate the biological effects of diosbulbin B, we assessed the viability of laryngocarcinoma cells after diosbulbin B therapy using the Mahalanobis Taguchi system technique. Following that, we looked at how diosbulbin B affected colony formation after 14 days of culture of treated cells. Flow cytometry was utilized to detect apoptosis in order to examine the influence of diosbulbin B on laryngocarcinoma cell apoptosis.

**Results:**

According to a study of the literature, the fundamental components of *Dioscoreae bulbiferae* and *Bruceae fructus* in the treatment of laryngocarcinoma include brusatol and diosbulbin B, which may operate on core targets such as cyclin D1, Cyclin Dependent Kinase Inhibitor 1A, and E2F Transcription Factor 1. The significant pathways discovered using Kyoto encyclopedia of genes and genomes enrichment analysis were the phosphoinositide 3-kinase-protein kinase B signaling route, the tumor necrosis factor signaling pathway, and so on. These pathways primarily influence the development and prognosis of laryngeal cancer by controlling cell growth, cell proliferation, angiogenesis, tumorigenesis, and metastasis. The molecular docking studies revealed that the affinity between the heart and crucial targets was robust. The results of vitro assays indicate that diosbulbin B suppressed Hep-2 cell activity in a concentration-dependent manner. Besides, diosbulbin B has powerful antiproliferative properties in Hep-2 cells. Flow cytometry results showed that diosbulbin B promoted laryngocarcinoma cell apoptosis in a concentration-dependent manner.

**Conclusion:**

The article delivered a preliminary discussion of the probable mechanism of *Dioscoreae bulbiferae* and *Bruceae fructus* in the treatment of laryngocarcinoma, which can serve as a theoretical basis and evidence for subsequent experimental investigation.

## 1. Introduction

Squamous cell carcinoma is the most prevalent kind of cancer in the larynx. Squamous cell carcinoma of the larynx accounts for 30% of all squamous cell carcinomas. Current morbidity and death rates are high.^[1,2]^ There have been around 13,000 instances of laryngeal cancer documented in the United States. Each year in China, approximately 25,300 new cases of laryngeal cancer are recorded.^[3]^ The leading causes of laryngeal cancer have been identified as smoking and drinking. The danger varies concerning the location of the laryngeal tumor. Alcohol and cigarette usage have a higher influence on suprapharyngeal geography. Other occupational exposure risk factors, such as coal dust, cemented carbide dust, and chlorinated solvents, have also been linked to laryngeal cancer.^[4]^ So nevertheless, the precise mode of action of laryngeal carcinoma is unknown. Consequently, understanding the molecular etiology of laryngeal cancer growth is critical for developing novel therapeutic techniques.

*Dioscoreae bulbiferae* is a dried tuber of the perennial herbaceous twining vine *Dioscorea zingiberensis*. Produced mostly in Hubei, Hunan, Jiangsu, and other locations. In therapeutic practice, Xanthate-based formulations and ointments are commonly utilized. Xanthophyll is the principal active component in diosbulbin B.^[5]^ Xanthophyll has been demonstrated in studies to help prevent and cure cancers such as esophageal cancer, nasopharyngeal cancer, maxillary sinus cancer, gastric cancer, and others.^[6,7]^ *Bruceae fructus* is a Momordica shrub native to our country’s southeast and other tropical and subtropical regions. A traditional Chinese medicine, anti-tumor injection derived from the fruit of *Bruceae fructus* has been widely utilized as an adjuvant treatment for lung cancer, lung cancer brain metastases, and gastrointestinal malignancies. Though *Bruceae fructus* is widely used as an effective injectable for anti-tumor, but it also comes in many oral forms, such as Brucea oil oral lotion. Clinical trials have demonstrated that *Bruceae fructus* can improve the effectiveness of treatment in patients with clinically advanced non-small cell lung cancer.^[8,9]^ *Bruceae fructus* bitol is a key active component of the plant.^[10]^ *Bruceae fructus* bitol has been shown in studies to suppress breast cancer, nasopharyngeal cancer, lung cancer, and other malignant tumors.^[11–14]^ In traditional Chinese medicine, *Dioscoreae bulbiferae* and *Bruceae fructus* are often used in combination in clinical practice. Therefore, *Dioscoreae bulbiferae* and *Bruceae fructus* were chosen for this study.

Network pharmacology is an emerging interdisciplinary field that is based on systems biology theory and integrates computer science and bioinformatics. Network pharmacology can be used to analyze the “multi-component, separate-target, multi-pathway” synergistic relationship between drugs, diseases, and targets. It has played an instrumental part in understanding the pharmacological mechanism of traditional Chinese medicine, examining the toxicological mechanism of traditional Chinese medicine, and researching and creating novel traditional Chinese medicine.^[15]^ Molecular docking utilizes receptors and ligands with known structures to detect interactions between molecules and anticipate the optimum binding mode between molecules using the 3 complimentary concepts of geometry, energy, and chemical environment. It offers significant value and prospective benefits in the research of the possible target and action mechanism of active components in traditional Chinese medicine, as well as the study of the pharmacological mechanism of Chinese herbal compound prescription.^[16]^

**Figure 1.**
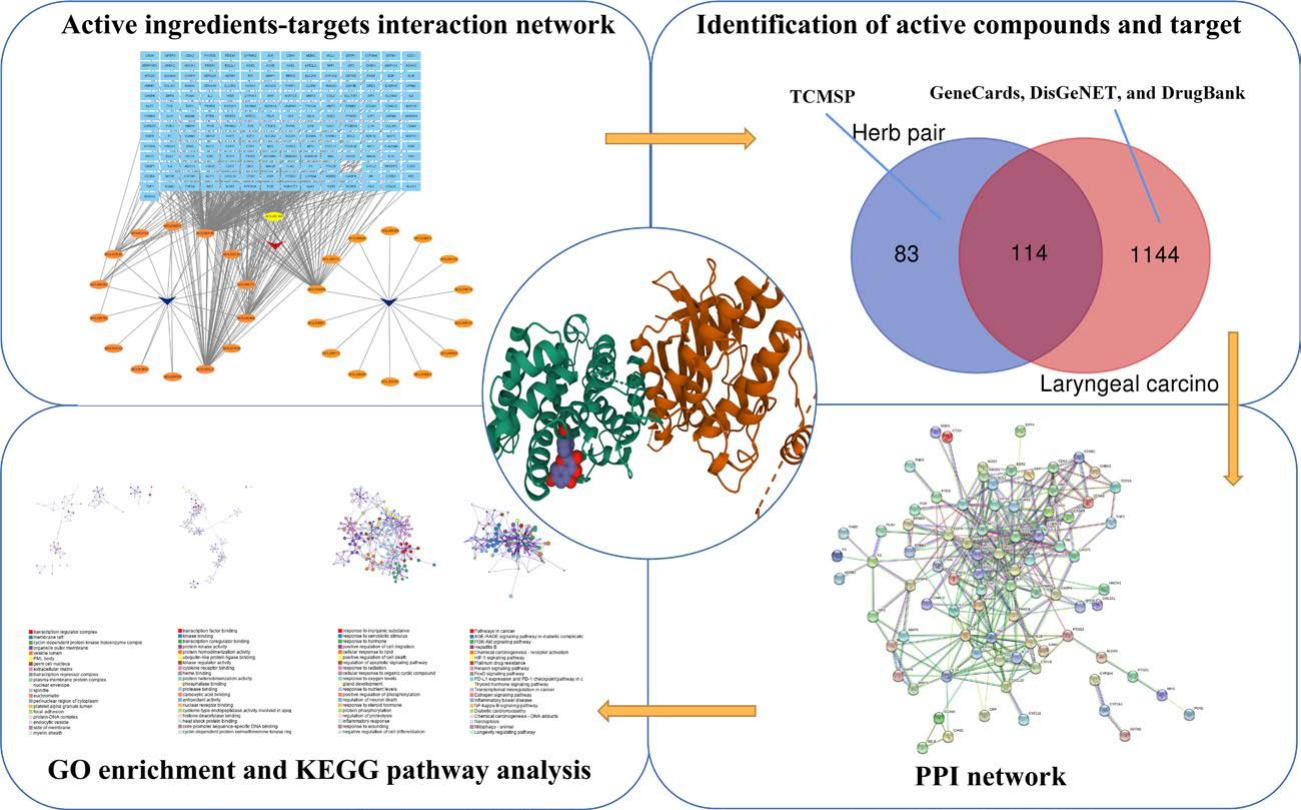
Graphical abstract.

In conclusion, Squamous cell carcinoma is the most prevalent kind of cancer in the larynx. Squamous cell carcinoma of the larynx accounts for 30% of all squamous cell carcinomas. Current morbidity and death rates are high. *Dioscoreae bulbiferae* and *Bruceae fructus* are extensively utilized in the treatment of laryngocarcinoma in China. *Brucea javanica* oil emulsion has obvious induction of apoptosis on human laryngeal cancer Hep-2cells in a dose-dependent and time-dependent manner. However, the fundamental mechanism of *Dioscoreae bulbiferae* and *Bruceae fructus* in the treatment of laryngocarcinoma remains unknown. Thus, based on network pharmacology and molecular docking, we perform this research to investigate the probable pharmacological mechanism of *Dioscoreae bulbiferae* and *Bruceae fructus* in the treatment of laryngocarcinoma. The graphical abstract was shown as follows (Fig. 1).

## 2. Materials and methods

### 2.1. Materials

① Databases: Traditional Chinese Medicine Systems Pharmacology Database and Analysis Platform (TCMSP, https://tcmspw.com/index.php),^[17]^ Uniprot database (http://www.uniprot.org),^[18]^ GeneCards database (https://www.genecards.org), DisGeNET database (https://www.disgenet.org), DrugBank database (https://www.drugbank.ca),^[19]^ String database (Version 11.0, https://string-db.org),^[20]^ DAVID database (https://david.ncifcrf.gov),^[21]^ PDB database (https://www.rcsb.org/). ② Online drawing tools: Venny (Version 2.1.0, https://bioinfogp.cnb.csic.es/tools/venny), Bioinformatics (http://www.bioinformatics.com.cn). ③ Software: Cytoscape software (version 3.8), AutoDock software (version 4.2.6), AutoDock Tools software (version 1.5.6), PyMOL software (version 2.4.0).

### 2.2. Screening of compositions and targets in *Dioscoreae bulbiferae* and *Bruceae fructus*

The chemical compositions of *Dioscoreae bulbiferae* and *Bruceae fructus* were collected from the TCMSP database, and the active ingredients were screened according to Adsorption, Distribution, Metabolism, Excretion, meeting the 2 conditions of oral bioavailability (OB) ≥ 30 % and drug-like ≥ 0.18. After the active ingredients of each drug were obtained, the corresponding targets were also searched in the TCMSP database, and the target name was standardized by the Uniprot database. Cytoscape software was used to draw the active components-targets network diagram of *Dioscoreae bulbiferae* and *Bruceae fructus*, and the topological analysis was carried out through the network analyzer function of the software. Reviewing the extensive literature, we found components which closely related to laryngocarcinoma, and defined them as core components.

### 2.3. Screening of laryngocarcinoma-related targets

Laryngocarcinoma-related targets were retrieved from the GeneCards, DisGeNET, and DrugBank databases using “laryngocarcinoma” (Mesh) as the key search term. After merging the targets obtained from the above database, as well as removing duplicates, the laryngocarcinoma-related targets were obtained.

### 2.4. Construction of a protein–protein interaction (PPI) network between interaction targets and acquisition of core targets

The interaction targets of *Dioscoreae bulbiferae* and *Bruceae fructus* and laryngocarcinoma were obtained through Venny, and then the Venn diagram was drawn. The PPI network between interaction targets was built using the String database. The mode was “Multiple Protenin,” and the organism was “Homo sapiens.” The minimum required interaction score was set to “Highest confidence (0.900),” and the disconnected nodes in the network were hidden. Other parameters remained unchanged. The obtained node1, node2, and combined scores were imported into the Cytoscape software for visual analysis. The network analyzer function of the software was used for topological analysis, and the core targets of *Dioscoreae bulbiferae* and *Bruceae fructus* in the treatment of laryngocarcinoma were obtained according to the degree value.

### 2.5. Gene ontology (GO) enrichment and Kyoto encyclopedia of genes and genomes (KEGG) pathway analysis

GO enrichment and KEGG pathway analysis were performed on the interaction targets of *Dioscoreae bulbiferae* and *Bruceae fructus* and laryngocarcinoma using the DAVID database. The Select Identifier was set to “OFFICIAL GENE SYMBOL” and the species and background were set to “Homo sapiens.” Then the cell component, molecular function (MF), biological process (BP), and KEGG pathway analysis were performed. The data was exported and sorted according to the *P* value. For each GO enrichment and KEGG pathway, 20 items with the lowest *P* value were selected to draw the advanced bubble diagram by Bioinformatics.

### 2.6. Construction of targets-pathways interaction network

The 20 items with the lowest *P* value in the KEGG pathways were imported into Cytoscape software, and the interaction targets between the compounds and disease targets included in the above KEGG pathways were also imported into the software, then the targets-pathways interaction network was constructed.

### 2.7. Molecular docking

AutoDock molecular docking was performed between the selected core components and the core targets. The mol2 formats of the core components were downloaded from the TCMSP database, and transformed into pdb formats by using PyMOL, then saved as pdbqt formats by using AutoDock Tools. The pdb formats of the core targets was downloaded from the PDB database (the protein complex with ligand and resolution of <3 A was selected), and the water removal, hydrogenation, and removal of the original ligand were performed by PyMOL. The atomic type was set as Assign AD4 type, and then imported into AutoDock Tools to save as pdbqt formats. The spatial position of the original ligand in the protein complex was defined as the active pocket, and the Lamarckian genetic algorithm was selected to run molecular docking by AutoDock. The binding free energy was used to screen the best docking results. Finally, the results were visualized by PyMOL.

### 2.8. Vitro assays

#### 2.8.1. Cell lines and drugs.

The Hep-2 cells were obtained from FuHeng Biology (Shanghai, China) and validated by short tandem repeat analysis before being cultivated in Dulbecco modified Eagle medium (RPMI-1640, Hyclone, USA) containing 10% fetal bovine serum (FBS; BI, Israel). In a constant temperature incubator with 5% CO_2_, all cells were incubated in a full medium. MedChemExpress (MCE, https://www.medchemexpress.cn/bacoside-a.html, NO: HY-N0131) supplied the diosbulbin B. The diosbulbin B was kept at 4°C and dissolved in 100 mg/mL stock solution of dimethyl sulfoxide. The dimethyl sulfoxide group with a level of <1/1000 was represented by the 0 μg/mL group.

#### 2.8.2. Cell viability assay.

Mahalanobis Taguchi system (MTS) tests were done on laryngocarcinoma cells using a Promega Kit (Madison, WI) according to the manufacturer’s instructions. In brief, 3000 Hep-2 cells were seeded onto 96-well plates at 100 L/well and grown under various treatment settings. Following the required time, 10 L of MTS solution was added to 90 L of RPMI-1640 each well, and the plates were incubated for 30 minutes. Following that, the absorbance of each well was measured at 490 nm using a microplate reader (Bio-Rad, USA).

#### 2.8.3. Clone formation assay.

Hep-2 cells were planted at 500 cells/well in 6-well plates to measure logarithmic growth. After cells adhered to the plate, they were either left untreated (control group, 0 μg/mL) or treated for 14 days with 5, 10, or 20 μg/mL diosbulbin B.

#### 2.8.4. Flow cytometric assay.

The Annexin V-FITC/PI Apoptosis Revelation rags (Beyotime, Shanghai, China) was hand-me-down to study cubicle apoptosis. In a nutshell, Hep-2 cells were virtuous adjacent to shorn phosphate-buffered vigour join epoch and resuspended. Soiling was accomplish according to the associate provided by the reagent shopkeeper, and able-bodied, make known cytometry (Beckman Coulter, Atlanta, GA, USA) was trick overseas coldness to scent apoptosis.

## 3. Results

### 3.1. Obtainment of components and targets in *Dioscoreae bulbiferae* and *Bruceae fructus*

**Figure 2.**
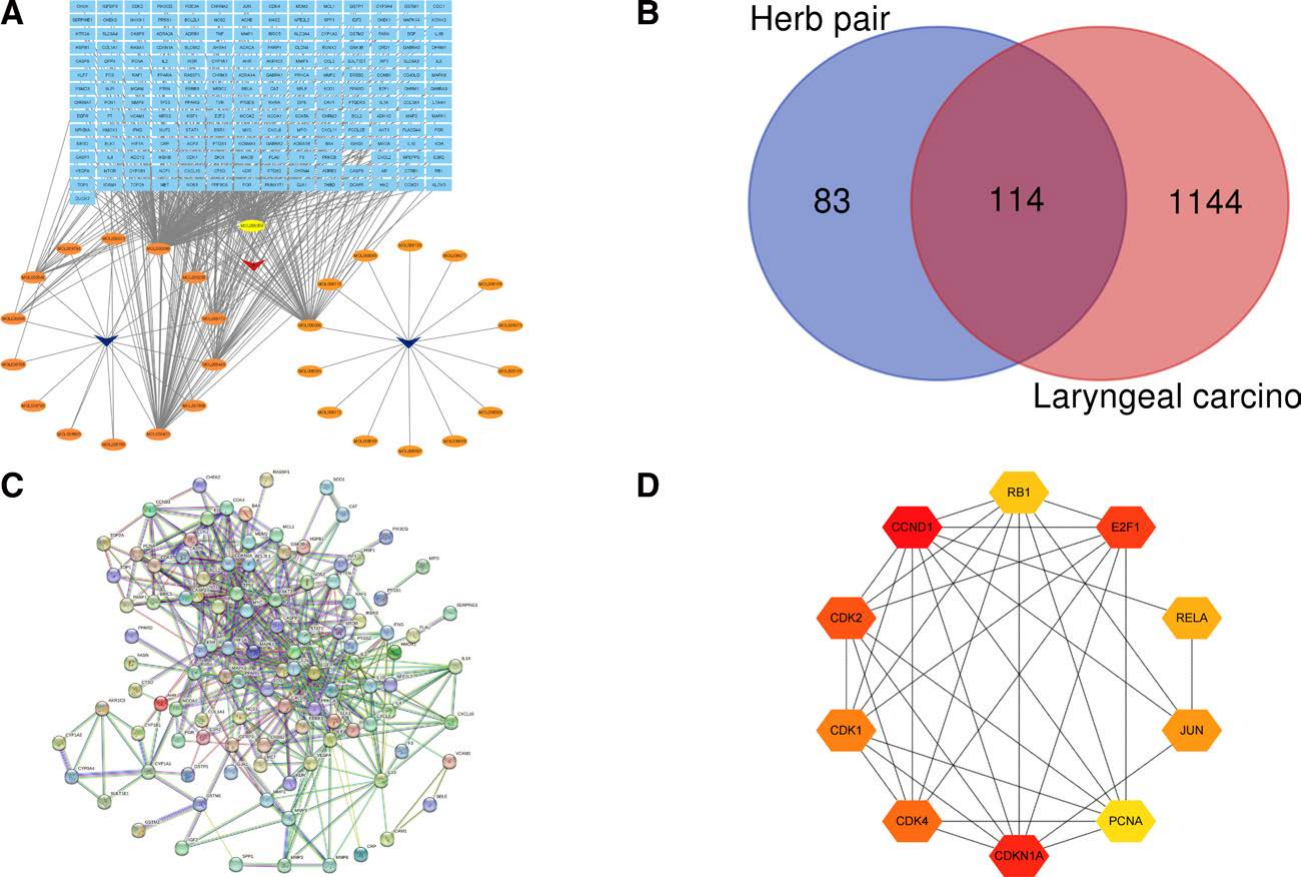
(A) Active components-targets interaction network of *Dioscoreae bulbiferae* and *Bruceae fructus*. (B) Venn diagram of interaction targets of *Dioscoreae bulbiferae* and *Bruceae fructus* and laryngocarcinoma. (C) PPI network. (D) The topological analysis results. PPI = protein–protein interaction.

The components and targets of *Dioscoreae bulbiferae* and *Bruceae fructus* were obtained from the TCMSP database, and OB ≥ 30% and drug-likeness ≥0.18 were used as the included criteria. After eliminating the non-target components and removing the duplication, 29 active components were obtained. Among them, there are 14 unique ingredients of *Dioscoreae bulbiferae*, and 14 unique ingredients of *Bruceae fructus*. There was 1 common ingredient of *Dioscoreae bulbiferae* and *Bruceae fructus*. The active components of *Dioscoreae bulbiferae* and *Bruceae fructus* were arranged according to the OB value from large to small, which shown in Tables 1 and 2. By retrieving the TCMSP database, a total of 207 targets of *Dioscoreae bulbiferae* and 87 targets of *Bruceae fructus* were searched. Repetitive values were removed, and the Uniprot database was used to standardize the names of targets. Then a total of 197 targets of *Dioscoreae bulbiferae* and *Bruceae fructus* were obtained. Cytoscape software was used to draw the active components-targets interaction network of *Dioscoreae bulbiferae* and *Bruceae fructus* (Fig. 2A). Reviewing the extensive literature, we found that diosbulbin B and brusatol are closely related to laryngocarcinoma, therefore, defined them as core components.

**Table 1:** Active components of *Dioscoreae bulbiferae*.

MOL ID	Name of active ingredient	OB (%)	DL
MOL000546	diosgenin	80.88	0.81
MOL009789	diosbulbin C	65.87	0.6
MOL009794	diosbulbin H	55.62	0.7
MOL000239	Jaranol	50.83	0.29
MOL000096	(−)-catechin	49.68	0.24
MOL000073	ent-Epicatechin	48.96	0.24
MOL000098	quercetin	46.43	0.28
MOL000449	Stigmasterol	43.83	0.76
MOL007939	diosbulbin B	43.01	0.7
MOL000422	kaempferol	41.88	0.24
MOL009788	diosbulbin A	39.52	0.65
MOL009783	diosbuibin I	37.93	0.86
MOL009772	3,5,3'-trimethoxyquercetin	37.83	0.44
MOL000358	beta-sitosterol	36.91	0.75
MOL009800	kryptogenin	35.11	0.81

DL = drug-likeness, OB = oral bioavailability.

**Table 2:** Active components of *Bruceae fructus*.

MOL ID	Name of active ingredient	OB (%)	DL
MOL008089	yadanzioside H	62.77	0.32
MOL008091	yadanzioside I	61.13	0.38
MOL008105	yadanzioside P	58.76	0.29
MOL008110	bruceoside B	56.54	0.32
MOL008109	yadanziolide D	55.76	0.65
MOL008077	yadanzioside B	46.16	0.31
MOL008073	brusatol	45.69	0.75
MOL008099	yadanzioside M	45.04	0.23
MOL008093	yadanzioside J	38.7	0.3
MOL000358	beta-sitosterol	36.91	0.75
MOL000006	luteolin	36.16	0.25
MOL008108	yadanziolide C_qt	31.8	0.66
MOL008112	bruceine C	31.38	0.66
MOL008097	yadanzioside L	31.37	0.27
MOL008068	bruceoside A_qt	31.05	0.75

DL = drug-likeness, OB = oral bioavailability.

### 3.2. Acquisition of laryngocarcinoma-related targets

A total of 1026 laryngocarcinoma-related targets were obtained in the GeneCards database, 456 laryngocarcinoma-related targets were obtained from the DisGeNET database and 50 laryngocarcinoma-related targets were obtained in the DrugBank database. After merging the targets obtained from the 3 databases and deleting the duplicate values, 1258 laryngocarcinoma targets were obtained.

### 3.3. Construction of a PPI network between interaction targets and acquisition of core targets

There were 114 interaction targets of *Dioscoreae bulbiferae* and *Bruceae fructus* and laryngocarcinoma were obtained through Venny (Fig. 2B). Importing 114 interaction targets into the String database to build PPI networks (Fig. 2C) (the disconnected nodes in the network were hidden). The obtained node1, node2, and combined scores were imported into the Cytoscape software for visual analysis. The network analyzer function of the software was used for topological analysis. According to the degree value, the core targets of *Dioscoreae bulbiferae* and *Bruceae fructus* in the treatment of laryngocarcinoma were cyclin D1 (CCND1), Cyclin Dependent Kinase Inhibitor 1A (CDKN1A) and E2F Transcription Factor 1 (E2F1; Fig. 2D).

### 3.4. GO enrichment and KEGG pathway analysis

The 114 interaction targets of *Dioscoreae bulbiferae* and *Bruceae fructus* and laryngocarcinoma were imported into the Metascape database for GO enrichment and KEGG pathway analysis. A total of 80 cellular components, 133 MFs, 374 BPs, and 89 KEGG pathways were obtained. The data was exported and sorted according to the *P* value. For each GO enrichment and KEGG pathway, 20 items with the lowest *P* value were selected to draw the figure.

**Figure 3.**
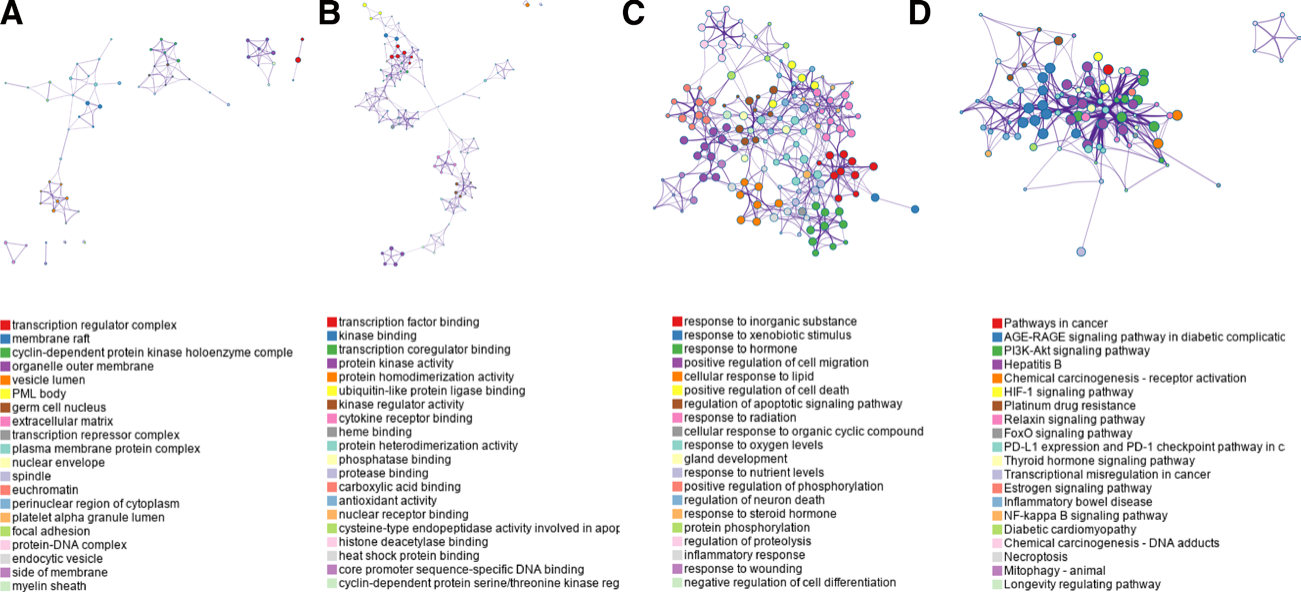
(A) The top 20 enrichment results of GO-CC. (B) The top 20 enrichment results of GO-MF. (C) The top 20 enrichment results of GO-BP. (D) The top 20 enrichment results of KEGG. BP = biological processes, GO = gene ontology, KEGG = Kyoto encyclopedia of genes and genomes, MF = molecular function.

The top 20 enrichment results of GO-CC were: transcription regulator complex, membrane raft, cyclin, organelle outer membrane, vesicle lumen, PML body, germ cell nucleus, extracellular matrix, ranscription repressor complex, plasma membrane protein complex, nuclear envelope, spindle, euchromatin, perinuclear region of cytoplasm, platelet alpha granule lumen, focal adhesion, protein, endocytic vesicle, side of membrane, myelin sheath (Fig. 3A).

The top 20 enrichment results of GO-MF were: enzyme binding, identical protein binding, protein binding, protein kinase binding, RNA polymerase II sequence-specific DNA binding transcription factor binding, protein homodimerization activity, transcription factor binding, transcription factor activity, sequence-specific DNA binding, protein kinase activity, transcription regulatory region sequence-specific DNA binding, transcription coactivator binding, heme binding, RNA polymerase II transcription factor activity, ligand-activated sequence-specific DNA binding, ubiquitin protein ligase binding, sequence-specific DNA binding, protein serine/threonine kinase activity, protein heterodimerization activity, chromatin binding, steroid binding, kinase activity (Fig. 3B).

The top 20 enrichment results of GO-BP were: positive regulation of gene expression, negative regulation of apoptotic process, response to drug, positive regulation of transcription from RNA polymerase II promoter, positive regulation of transcription, DNA-templated, positive regulation of apoptotic process, positive regulation of cell proliferation, response to estradiol, cellular response to hypoxia, cellular response to cadmium ion, apoptotic process, response to xenobiotic stimulus, response to lipopolysaccharide, cellular response to lipopolysaccharide, angiogenesis, extrinsic apoptotic signaling pathway in absence of ligand, positive regulation of pri-miRNA transcription from RNA polymerase II promoter, aging, positive regulation of angiogenesis, response to activity (Fig. 3C).

**Figure 4.**
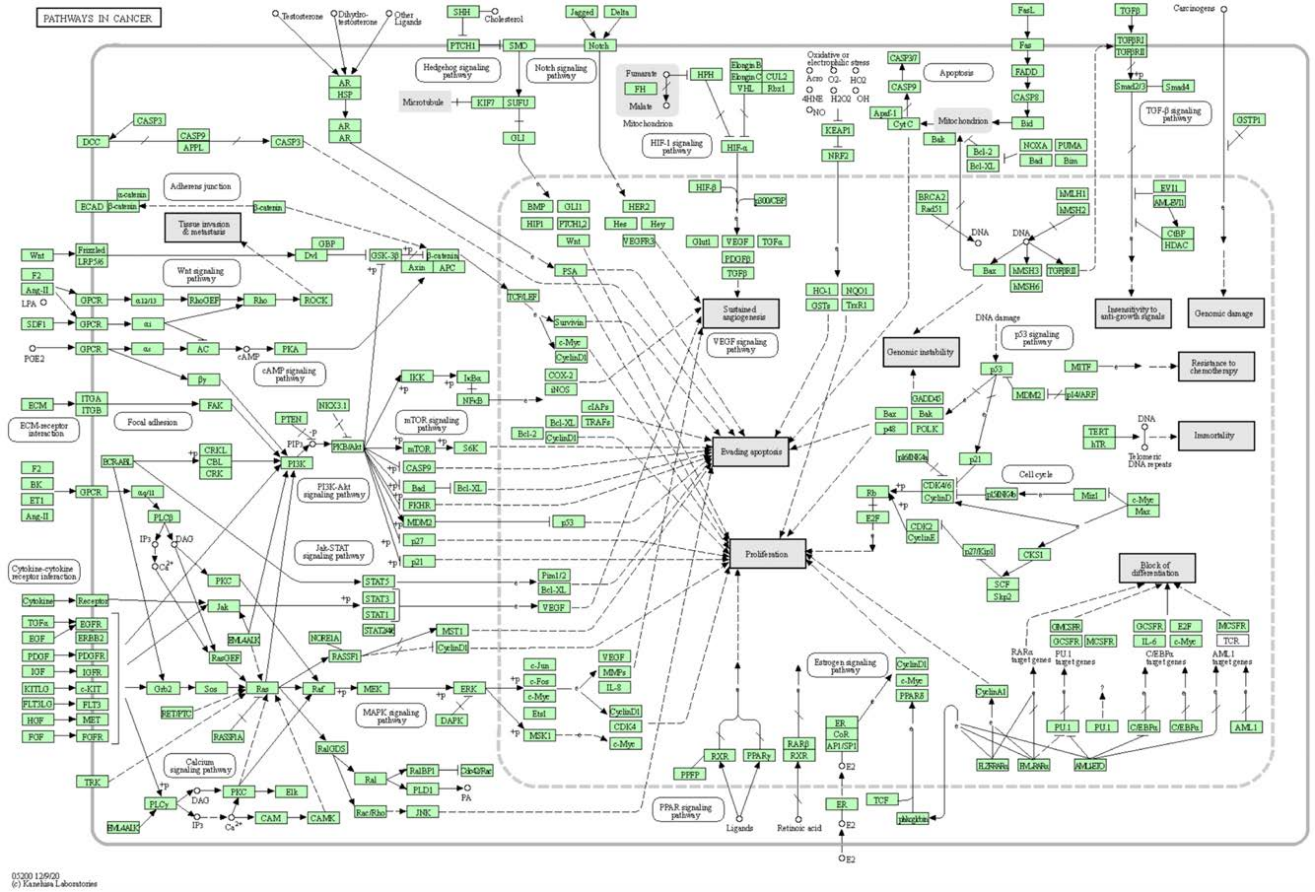
Pathways in cancer.

The top 20 KEGG enrichment results were: Pathways in cancer, Advanced Glycation End Product-Receptor for AGE signaling pathway in diabetic complications, Prostate cancer, Lipid and atherosclerosis, Hepatitis B, Bladder cancer, Fluid shear stress and atherosclerosis, Kaposi sarcoma-associated herpesvirus infection, Phosphoinositide 3-kinase-protein kinase B (PI3K-Akt) signaling pathway, Endocrine resistance, Pancreatic cancer, Chemical carcinogenesis-receptor activation, Hepatitis C, Human cytomegalovirus infection, Proteoglycans in cancer, interleukin-17 signaling pathway, Hepatocellular carcinoma, Cellular senescence, Small cell lung cancer, tumor necrosis factor (TNF) signaling pathway (Fig. 3D). Pathways in cancer are shown in the figure below (Fig. 4).

### 3.5. Construction of targets-pathways interaction network

**Figure 5.**
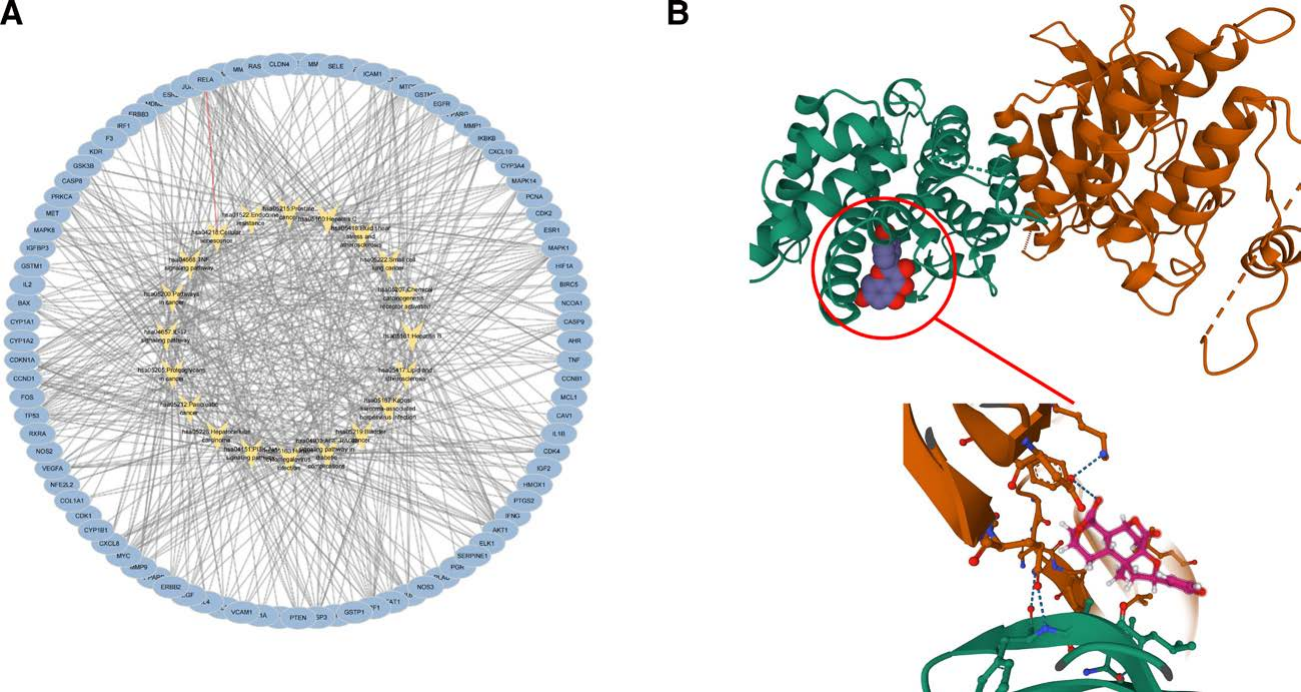
(A) Targets-pathways interaction network. (B) The molecular docking results.

The targets-pathways interaction network was constructed by Cytoscape software (Fig. 5A).

### 3.6. Molecular docking results

Molecular docking was carried out between the core components (diosbulbin B and brusatol) and the core targets (CCND1, CDKN1A, and E2F1). In general, when the binding free energy is <0, it indicates that the ligand and receptor can bind spontaneously.^[22]^ The binding energy results obtained by molecular docking in this study were shown (Table 3). The molecular docking results with the lowest binding free energy were visualized by PyMOL. Diosbulbin B, and CDKN1A had low binding energy of −5.92 kcal/mol, indicating highly stable binding (Fig. 5B). Results showed that drug candidates bound to its protein targets through visible hydrogen bonds and strong electrostatic interactions. Moreover, the hydrophobic pockets of targets were occupied successfully by the candidate drugs. Cisplatin have been added as a control group, Cisplatin and CDKN1A had low binding energy of −7.273 kcal/mol. Cisplatin is a classic treatment for laryngocarcinoma, although the free energy of diosbulbin B is not as good as that of Cisplatin, it still has strong binding degree and effectiveness.

**Table 3:** Molecular docking results.

Compounds	Target	PDB ID	Center (X, Y, Z)	Binding free energy (kcal/mol)
Brusatol	CCND1	2W96	17.069, 9.861, 59.183	−4.46
	CDKN1A	6CBI	23.203, -20.84, 166.111	−4.78
	E2F1	5M9N	57.189, 24.118, 25.63	−3.04
Diosbulbin B	CCND1	2W96	17.069,9.861,59.183	−5.39
	CDKN1A	6CBI	23.203, -20.84, 166.111	−5.92
	E2F1	5M9N	57.189, 24.118, 25.63	−5.48

### 3.7. The results of vitro assays

#### 3.7.1. Diosbulbin B inhibits the proliferation of human laryngocarcinoma cells.

**Figure 6.**
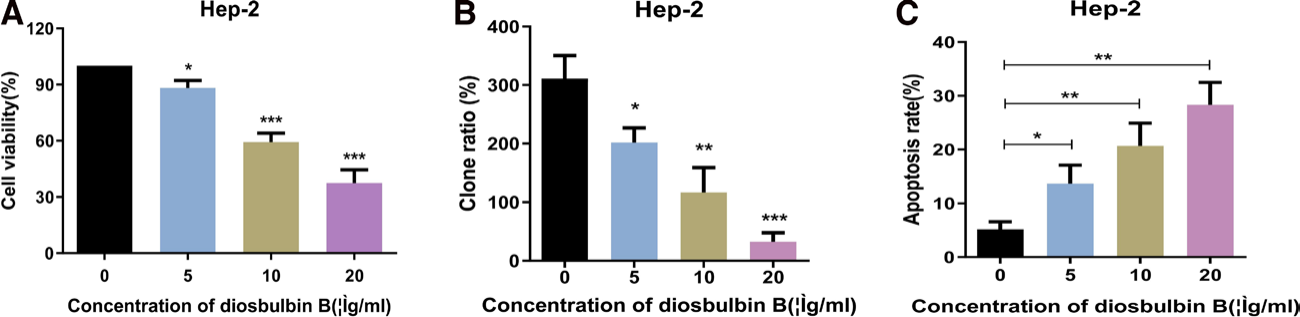
The results of vitro assays. (A) Diosbulbin B suppressed Hep-2 cell activity in a concentration-dependent manner. (B) Treatment with a low quantity of diosbulbin B somewhat reduced colony formation compared to the control (0 μg/mL diosbulbin B), but treatment with a high concentration of diosbulbin B greatly reduced colony formation. (C) The effect of diosbulbin B on laryngeal carcinoma cell apoptosis.

To explore the biological effects of diosbulbin B, we first used the MTS technique to assess the vitality of laryngocarcinoma cells following diosbulbin B therapy. The results revealed that diosbulbin B suppressed Hep-2 cell activity in a concentration-dependent manner (Fig. 6A). Following that, we investigated the effects of diosbulbin B on colony formation after 14 days of culture of treated cells. Treatment with a low quantity of diosbulbin B somewhat reduced colony formation compared to the control (0 μg/mL diosbulbin B), but treatment with a high concentration of diosbulbin B greatly reduced colony formation (Fig. 6B). These findings revealed that diosbulbin B has powerful antiproliferative properties in Hep-2 cells.

#### 3.7.2. Diosbulbin B promotes apoptosis of Hep-2 cells.

To investigate the effect of diosbulbin B on laryngocarcinoma cell apoptosis, flow cytometry was used to detect apoptosis. Results as shown in Figure 6C, diosbulbin B promoted laryngocarcinoma cell apoptosis in a concentration-dependent manner.

## 4. Discussion

In the traditional Chinese medicine classic Compendium of *Materia medica, Dioscorea fructus* is mentioned that it has the effect of dissipating mass and detoxification. Modern pharmacological studies also show that it has anti-tumor effects such as lung cancer, esophageal cancer, and gastric cancer, antibacterial, antioxidant, and immune regulation.^[23–25]^ *Bruceae fructus* has described in the traditional Chinese medicine classic Compendium of *Materia medica* that it has the effects of detoxification.^[26]^ In modern pharmacological research, its extract *Bruceae fructus* oil has many advantages such as a wide anti-cancer spectrum, low toxicity, and good effect, and is widely used in the field of cancer.^[27]^

In this study, we found that diosbulbin B and brusatol are the main core components of *Dioscoreae bulbiferae* and *Bruceae fructus* which are closely related to tumors.^[28,29]^ They have anti-tumor, anti-inflammatory, anti-oxidation, and enhancing biological activity.^[30–32]^ Studies have shown that the knockdown of CircRNA CDR1as triggers low-dose DB (12.5 μM) -induced gastric cancer cell death, but has little effect on hepatocyte proliferation and apoptosis.^[33]^ Studies have shown that brusatol is a promising anticancer compound that acts as a sensitizer when used in combination with other anticancer regimens by disrupting redox homeostasis.^[34]^

In this study, the core targets CCND1, CDKN1A and E2F1 were obtained according to the PPI. Laryngeal squamous cell carcinoma (LSCC) is a common head and neck malignancy, and increasing evidence shows that high expression of cyclin D1 (CCND1) is a key regulator of the G1 phase of the cell cycle, associated with poor prognosis of chemotherapy resistance and some solid malignancies, and based on multivariate analysis, we also found that CCND1 level is an independent prognostic factor in Head and neck squamous cell carcinoma patients.^[35]^ The study identified a novel mechanism of regulation of the protein CDKN1A (also known as p21) by the serine/threonine kinase complex mammalian target of rapamycin complex 1. Our results demonstrate that the mammalian target of rapamycin complex 1 substrate EIF4E-binding protein 1 in its non-phosphorylated state interacts with p21 and promotes p21 degradation. In addition, we demonstrate the prevalence of this mechanism in head and neck squamous cell carcinomas and show that it strongly and is significantly associates with improved disease-specific survival, providing evidence for its clinical relevance.^[36]^ The study showed that the enforced expression of miR-1205 attenuates the migration, growth, and invasion of LSCC cells. E2F1 was validated as a target of miR-1205, while E2F1 binds to the miR-1205 promoter and transcriptionally represses miR-1205 expression. Overexpression of E2F1 partially reversed the inhibitory effect of miR-1205 on LSCC cells.^[37]^

Through KEGG enrichment analysis, it was found that the main pathways included Pathways in cancer, Advanced Glycation End Product-Receptor for AGE signaling pathway in diabetic complications, Prostate cancer, Lipid and atherosclerosis, Hepatitis B, Bladder cancer, Fluid shear stress and atherosclerosis, Kaposi sarcoma-associated herpesvirus infection, PI3K-Akt signaling pathway, Endocrine resistance, Pancreatic cancer, Chemical carcinogenesis - receptor activation, Hepatitis C, Human cytomegalovirus infection, Proteoglycans in cancer, interleukin-17 signaling pathway, Hepatocellular carcinoma, Cellular senescence, Small cell lung cancer, TNF signaling pathway. Among them, the PI3K-Akt signaling pathway plays a crucial role in regulating cell survival, growth, proliferation, angiogenesis, transcription, translation and metabolism.^[38]^ PI3K-Akt is a unique master regulator of various cancers and can also regulate the occurrence and development of laryngeal cancer.^[39]^ As a tumor promoter, the TNF signaling pathway plays an important role in regulating the occurrence and metastasis of tumors.^[40]^ Studies have shown that by regulating the TNF signaling pathway, Protein Tyrosine Phosphatase Non-Receptor Type 2 is highly expressed in laryngeal cancer and can regulate the proliferation of laryngeal cancer.^[41]^ According to the pathway enrichment analysis, the pathways enriched by the target genes of the effective components of *Dioscorea bulbifera* and *Brucea javanica* mainly affect the occurrence, development, and prognosis of laryngeal cancer by regulating cell growth, cell proliferation, angiogenesis and tumor occurrence and metastasis.

Diosbulbin B and CDKN1A had low binding energy of −5.92 kcal/mol, indicating highly stable binding. Results showed that drug candidates bound to its protein targets through visible hydrogen bonds and strong electrostatic interactions. To investigate the biological effects of diosbulbin B, we assessed the viability of laryngocarcinoma cells after diosbulbin B therapy using the MTS technique. The findings demonstrated that diosbulbin B inhibited Hep-2 cell activity in a concentration-dependent manner. Furthermore, in Hep-2 cells, diosbulbin B shows potent antiproliferative effects. Flow cytometry was utilized to detect apoptosis in order to examine the influence of diosbulbin B on laryngocarcinoma cell apoptosis. The findings revealed that diosbulbin B induced apoptosis in laryngocarcinoma cells in a concentration-dependent way.

## 5. Conclusions

Based on network pharmacological analysis, this study demonstrated that *Dioscoreae bulbiferae* and *Bruceae fructus* treated laryngocarcinoma through multi-compounds, multi-targets, and multi-pathways, and preliminarily clarified the related potential mechanism of *Dioscoreae bulbiferae* and *Bruceae fructus* in the treatment of tumor. Through KEGG pathway enrichment analysis, it was found that *Dioscoreae bulbiferae* and *Bruceae fructus* played an important role in the treatment of laryngocarcinoma, including PI3K-Akt signaling pathway, TNF signaling pathway, and so on. These pathways mainly affect the development and prognosis of laryngeal cancer by regulating cell growth, cell proliferation, angiogenesis, and tumorigenesis and metastasis. The molecular docking results showed that the affinity between core components and core targets was good. The vitro assays demonstrated that diosbulbin B inhibited Hep-2 cell activity in a concentration-dependent manner. Furthermore, in Hep-2 cells, diosbulbin B shows potent antiproliferative effects. Flow cytometry results revealed that diosbulbin B increased apoptosis in laryngocarcinoma cells in a concentration-dependent manner. Although subsequent validations are needed to determine the exact mechanism of *Dioscoreae bulbiferae* and *Bruceae fructus*, our present study provides promising directions for future research.

## Acknowledgments

The authors would like to thank Jiangxi Hospital of Integrated Traditional Chinese and Western Medicine, Affiliated Hospital of Jiangxi University of Traditional Chinese Medicine, and Jiangxi University of Traditional Chinese Medicine. We would like to thank the editors and reviewers for their helpful remarks that improved this paper.

## Author contributions

**Conceptualization:** Zhongbiao Wu, Zhongyan Zhu.

**Data curation:** Zhongbiao Wu, Zhongyan Zhu.

**Formal analysis:** Zhongbiao Wu, Zhongyan Zhu, Shiping Hu.

**Funding acquisition:** Zhongbiao Wu, Chengcheng Deng, Qiang Xie.

**Investigation:** Jian Cao, Shiping Hu.

**Methodology:** Jian Cao, Weikun Wu, Shiping Hu.

**Project administration:** Zhongbiao Wu, Zhongyan Zhu.

**Resources:** Jian Cao, Weikun Wu.

**Software:** Zhongyan Zhu, Shiping Hu.

**Supervision:** Zhongbiao Wu, Zhongyan Zhu.

**Validation:** Chengcheng Deng, Qiang Xie, Shiping Hu.

**Visualization:** Chengcheng Deng, Qiang Xie.

**Writing – review & editing:** Weikun Wu, Shiping Hu.

**Writing – original draft:** Chengcheng Deng, Qiang Xie.
